# Transcranial pulsed electromagnetic fields for treatment-resistant depression: A multicenter 8-week single-arm cohort study: The eighth trial of the Danish University Antidepressant Group

**DOI:** 10.1192/j.eurpsy.2020.3

**Published:** 2020-02-18

**Authors:** Erik Roj Larsen, Rasmus W. Licht, René Ernst Nielsen, Annette Lolk, Bille Borck, Claus Sørensen, Ellen Margrethe Christensen, Gustav Bizik, Janus Ravn, Klaus Martiny, Maj Vinberg, Odeta Jankuviené, Pernille Blenker Jørgensen, Poul Videbech, Per Bech

**Affiliations:** 1 Mental Health Department Odense, University Clinic, Mental Health Service, Odense, Denmark; 2 Department of Clinical Research, University of Southern Denmark, Odense, Denmark; 3 Aalborg University Hospital—Psychiatry, Aalborg, Denmark; 4 Department of Clinical Medicine, Aalborg University, Aalborg, Denmark; 5 Centre for Neuropsychiatric Depression Research, Mental Health Centre Glostrup, Glostrup, Denmark; 6 Department of Affective Disorders, Aarhus University Hospital, Skejby, Denmark; 7 Copenhagen Affective Disorder Research Centre (CADIC), Psychiatric Centre Copenhagen, Copenhagen University Hospital, Copenhagen, Denmark; 8 Psychiatric Research Unit, Mental Health Centre North Zealand, University of Copenhagen, Copenhagen, Denmark

**Keywords:** major depressive disorder, transcranial pulsed electromagnetic fields, treatment-resistant depression

## Abstract

**Background.:**

The efficacy of antidepressant treatment is fair, but the efficacy is considerably lower in patients failing two or more trials underscoring the need for new treatment options. Our study evaluated the augmenting antidepressant effect of 8-weeks transcranial pulsed electromagnetic field (T-PEMF) therapy in patients with treatment-resistant depression.

**Methods.:**

A multicenter 8-week single-arm cohort study conducted by the Danish University Antidepressant Group.

**Results.:**

In total, 58 participants (20 men and 38 women) with a moderate to severe depression as part of a depressive disorder according to ICD-10 who fulfilled criteria for treatment resistance were included, with 19 participants being nonresponders to electroconvulsive therapy during the current depressive episode. Fifty-two participants completed the study period. Scores on the Hamilton Depression Scale 17-items version (HAM-D_17_) decreased significantly from baseline (mean = 20.6, SD 4.0) to endpoint (mean = 12.6, SD 7.1; *N* = 58). At endpoint, utilizing a Last Observation Carried Forward analysis, 49 and 28% of those participants with, respectively, a nonchronic current episode (≤2 years; *N* = 33) and a chronic current episode (>2 years; *N* = 25) were responders, that is, achieved a reduction of 50% or more on the HAM-D_17_ scale. At endpoint, respectively, 30 and 16% obtained remission, defined as HAM-D_17_ ≤ 7. On the Hamilton Scale 6-item version (HAM-D_6)_, respectively, 51 and 16% obtained remission, defined as HAM-D_6_ ≤ 4.

**Conclusions.:**

The findings indicate a potential beneficial role of T-PEMF therapy as an augmentation treatment to ongoing pharmacotherapy in treatment-resistant depression.

## Introduction

The efficacy of antidepressant treatment is fair, but the efficacy is considerably lower in patients failing two or more trials [1]. The proportion of remitters decreases dramatically after the second failed treatment trial. The cumulative proportion of remitters was estimated to 67% after four trials in the STAR*D study [2]. Furthermore, the likelihood of antidepressant treatment effect decreases with the duration of the current depressive episode [3], with episodes shorter than 12 months being associated with cumulative proportions of remitters of 80–90%, whereas episodes of longer duration than 12 months are associated with a 40% cumulative proportion of remitters [4, 5] for both pharmacological and electroconvulsive therapy (ECT) [6]. Although no consensus concerning the definition of treatment resistant depression (TRD) exists, it is usually defined as insufficient effect within the same episode of at least two different evidence-based medical treatments, that is, two antidepressants from different classes evaluated over a sufficient time period (at least 6 weeks) and given in a sufficient dose [7].

Treatment with transcranial pulsed electromagnetic fields (T-PEMFs) for patients with otherwise treatment-resistant depression has previously been examined with promising results [8].

The treatment helmet consists of seven electromagnetic coils. It is placed on the head of the patient and is connected to an impulse generator resulting in a supposed diffuse, multifocal brain stimulation of several brain regions. The patient is awake during treatment. T-PEMF induces low electric fields near 0.0004 V/m compared with repetitive transcranial magnetic stimulation (rTMS), which results in 90 V/m. In contrast to rTMS, T-PEMF stimulation stays well below the firing threshold of neurons, but may modify intrinsic brain oscillation [9, 10]. The Re5 PEMF pulse generator powers the coils with alternating bipolar square pulses, each lasting 3 ms and interspersed by a 12-ms pause, each pulse sequence thus lasting 18 ms, corresponding to a pulse frequency of 55 Hz. Interactions of low frequency pulsed electromagnetic fields with living tissue has been reviewed by Dissing et al. [10]. They concluded that a general picture emerges from studies on cell cultures and living tissue revealing that PEMF facilitates existing biochemical processes—especially those related to growth factor stimulation. Most of these factors stimulate cytoplasmic tyrosine kinases of the Src family and they lead to many different responses depending on the cell type, that is, proliferation of osteoblasts, endothelial cells, nerve cells, and chondrocytes.

In a double-blind, randomized, sham-controlled study, rTMS was shown to reduce HAM-D_17_ scores from 25.3 (SD 3.0) to 11.1 (SD 6.7) after 12 weeks in the active treatment group versus 24.7 (SD 3.2) to 13.5 (SD 7.2) in the sham group (*p* < 0.22) [11]. Participants randomized to rTMS were treated over 3 weeks at a psychiatric research unit and followed-up for 9 more weeks. Similarly, T-PEMF was investigated in a double-blind, randomized, sham-controlled study [8]. In this study, HAM-D_17_ scores were reduced from 21.1 (SD 4.1) to 11.0 (SD 5.7) after 5 weeks in the active treatment group versus 20.9 (SD 3.3) to 16.0 (SD 5.6) in the sham group (*p* < 0.01). The response proportion was 61.0% in the T-PEMF group versus 12.9% in the sham group, with remission proportion of 33.9% versus 4.1%, respectively. The low remission proportion in the placebo group in this study population of TRD patients is similar to previous studies [12]. Furthermore, a double-blind, randomized controlled study comparing once or twice daily T-PEMF treatment for 8 weeks administered in the patients’ homes on all weekdays showed a reduction in HAM-D_17_ from 20.4 (SD 2.7) to 6.8 (SD 4.5) and from 20.9 (SD 2.9) to 7.3 (SD 5.8); *p* < 0.92. The proportion of remitters (defined as HAM-D_17_ ≤ 7) was 73.5 and 67.7%, respectively (*p* = 0.79) [13].

In the 2-year follow-up of the study mentioned [13], looking at the 73.5% of patients who obtained remission during the acute phase treatment, 48% had a relapse between 4 and 16 months after the T-PEMF augmentation [14] and remission was achieved after a repeated course of T-PEMF. No serious side effects were experienced in the mentioned T-PEMF studies, besides a minor headache and slight nausea after finishing the first treatments.

Besides symptom reduction, the patient’s quality of life is important. In the first T-PEMF study [8], the WHO-5 well-being scale was utilized to measure quality of life, which increased from baseline to endpoint. This difference was, however, not statistically significant. WHO-5 was also used in the present study.

We have included the HAM-D_6_ subscale as well as it consists of the core symptoms of depression (depressed mood, guilt, work and interests, psychomotor retardation, psychic anxiety, and general somatic). The subscale has been found to perform better than the full HAM-D_17_ scale in term of unidimensionality [15, 16]. The HAM-D_17_ scale also measures side effects induced by antidepressants such as sleep disturbances, agitation, gastrointestinal symptoms, and sexual disturbances.

## Aims of the study

The current study aimed at evaluating whether an 8-week T-PEMF treatment used as an augmentation to ongoing pharmacological antidepressant treatment in patients with treatment-resistant depression in a multicenter, single-arm study design would replicate the findings of previous studies obtained at only one research center. Furthermore, we aimed at evaluating if duration of the current depressive episode was associated with treatment response by comparing nonchronic (≤2 years) with chronic current episode (>2 years). Lastly, we wanted to evaluate whether ECT nonresponders were able to achieve response by T-PEMF, as the two treatments seem to work differently.

## Materials and Methods

### Design

An 8-week single-arm cohort study augmenting treatment as usual with daily T-PEMF treatments.

### Study population

Adult patients (≥18 years) were screened from the six participating mental health centers (Table 1). A checklist was filled in with the inclusion and exclusion criteria with an accept from the potentially eligible patients who wanted to be contacted for more information. Inclusion and exclusion criteria were then confirmed, and outcome measures at baseline and other relevant information were recorded by the study team after informed consent to participate was obtained. Participants who were not referred to the project were left unregistered.

**Table 1.** Inclusion according to centers and characteristics of all participants with treatment resistant depression at baseline and stratified according to duration of depression

	All participants	Participants with a duration of depression (≤2 years)	Participants with a duration of depression (>2 years)
Centers			
Aalborg	17	13	4
Risskov	12	6	6
Odense	8	6	2
PC Glostrup	8	2	6
PCK—KBH	10	3	7
PCN—Hillerod	3	3	0
Total	58	33	25
Females	66%	64%	68%
Age mean (years)	53.7 (SD 14.9)	54.2 (SD 13.6)	52.2 (SD 16.8)
Mild depression	14%	21%	4%
Moderate depression	55%	64%	44%
Severe depression	31%	15%	52%
Number of episodes	4.4 (SD 4.2)	4.8 (SD 4.2)	4.0 (SD 4.3)
Mean HAM-D17	20.6 (SD 4.0)	19.3 (SD 3.9)	22.3 (SD 4.2)
Mean HAM-D6	11.2 (SD 2.4)	10.6 (SD 2.5)	12.1 (SD 1.9)
Mean WHO-5	19.9 (SD 15.9)	23.0 (SD 17.7)	15.8 (SD 12.2)

Inclusion criteria were a diagnosis of moderate to severe depression, single episode or recurrent according to ICD-10 (F32 and F33), and with a Hamilton Depression Scale score of 13 or more on the 17 item version (HAM-D_17_) [15] and further fulfilling the criteria for treatment-resistant depression characterized by an insufficient effect of at least two different evidence-based medical treatments, that is, two antidepressants from different classes, and evaluated over a sufficient amount of time (at least 6 weeks) and given in a sufficient dose [17]. Both hospitalized and outpatients were included. ECT nonresponders could be included in the project if they fulfilled the criteria of no response to two antidepressants as mentioned above. Participants with comorbid anxiety disorders were included.

Exclusion criteria were a diagnosis of dementia, schizophrenia, bipolar disorder, borderline personality disorder, harmful use or dependence of psychoactive drugs, or severe suicidality, defined as HAM-D_17_, item 3 (suicidal ideations) score of >2, as well as severe physical disease (e.g., cancer, leukemia, melanoma or other cancer forms in neck or head, autoimmune diseases, organic diseases in the brain, Parkinson’s disease, multiple sclerosis, epilepsy, stroke, or use of pacemaker) interfering with study participation, as judged by the investigator. Participants unable to give informed consent were excluded.

The pharmacological treatment had to remain unchanged over the last 4 weeks before the initiation of the T-PEMF as well as during the study period. However, initiation or changes in sleep aid medication were allowed, for example, benzodiazepines, imoclone, zopiclone, melatonin, and promethazine. As participants were followed-up at the hospital or outpatient clinic, we were able to secure that no change of medication was done by getting access to data concerning the medication used. Besides, participants were informed that no change of medication was allowed during T-PEMF treatment.

The study was planned to be prematurely terminated if adverse events occurred that were more serious than expected or if new evidence was found during the treatment period that indicated the treatment should be regarded as a less safe treatment. In case of rapid and severe deterioration of depressive symptoms requiring ECT or change in psychopharmacological treatment or if mania occurred, the participant was withdrawn from the study.

### Outcome measures

Change in the Hamilton Depression Scale (HAM-D_17_) was chosen as the primary outcome measure. Secondary outcome measures were response and remission on the HAM-D_17_. HAM-D_17_ consists of 17 items, which can be scored between 0 and 2 or 0 and 4, resulting in a total score between 0 and 52 [18]. Remission was defined as a HAM-D_17_ score at the endpoint of seven or less, and response was defined as a 50% reduction or more on the HAM-D_17_ total score at endpoint. Response and remission on the Hamilton Depression Scale 6 item version (HAM-D_6_) were evaluated as well with response defined as at least a 50% reduction from baseline in total score and remission defined as a HAM-D_6_ score ≤4. The HAM-D_6_ subscale includes the core symptoms of depressive states (depressed mood, guilt, work and interests, psychomotor retardation, psychic anxiety, and general somatic) [15]. Inter-rater reliability was secured in different ways. The Danish University Antidepressant Group (DUAG) has produced three videos in Danish for Hamilton inter-rater training. Besides, the HAM-D_17_ is used on a weekly basis in hospital-based psychiatric settings in Denmark, as all patients treated for depression must be rated with HAM-D_17_ before and after treatment. The data are delivered to the Danish National Clinical Quality Development Program. In addition, each study participant was rated by the same rater throughout the study.

Side effects were evaluated utilizing the self-reported Patient Reported Inventory of Side Effects (PRISE) [15]. It consists of 11 items scored from 0 to 2 according to the severity of side effects.

The WHO-5 well-being scale [15] was utilized to measure quality of life. WHO-5 contains only five positive phrased items to measure positive psychological well-being. Each item is scored in terms of frequency over the past 2 weeks. The score on each item has a range from 0 (none of the time) to 5 (all the time). The summed total score of the WHO-5 goes from 0 (no well-being) to 25 (extreme well-being), with the sum score being multiplied by four, to result in a score from 0 to 100.

### Statistical analyses

The sample size calculation was based on the two previous T-PEMF studies [8, 13]. Using a two-sided paired *t*-test with a significance level of 0.05 and a power of 0.90 to detect a meaningful difference of 4 points from baseline to endpoint on the HAM-D_17_ would require at least 26 participants. With an expected dropout of 15%, the minimum sample size would be 31 participants.

Initially, a descriptive analysis of the study population’s baseline characteristics was conducted.

Second, baseline scores of HAM-D_17_ and HAM-D_6_ were compared with scores at endpoint for each patient using paired *t*-tests, that is, intention-to-treat analyses. *T*-tests for independent samples were used to compare the mean scores of HAM-D_17_ and HAM-D_6_ between participants with nonchronic (defined as duration of the current depressive episode of 2 years or less) and participants with chronic current depressive episode (defined as duration of the current depressive episode of more than 2 years) at baseline and at 2-, 4-, 6- and 8-weeks’ follow-up. Also, the proportion of responders (response defined as ≥50% reduction in HAM-D score from baseline until endpoint), proportion of remitters (remission defined as HAM-D_17_ ≤ 7 [or HAM-D_6_ ≤ 4] at endpoint), and the proportions of participants with a WHO-5 score of at least 50% at endpoint were compared between participants with chronic and nonchronic current depressive episode using Pearson’s chi-squared tests. Response and remission analyses were performed both as complete case analyses and as intention-to-treat analyses (endpoint-analysis, which essentially is the same as Last Observation Carried Forward analysis).

### Intervention

Both hospitalized and outpatients were included. Participants were given the first T-PEMF treatment at the hospital or outpatient clinic and were instructed about the use of T-PEMF. Thereafter, they were supervised per need at the hospital or outpatient clinic during the first week to ensure proper use. After instruction, treatment was self-administered in the participant’s home. Duration of treatment was 30 min per day, each day, with treatments for 8 weeks. All instructors were qualified to use T-PEMF by Re5 ApS, Denmark. The participants were thereafter seen at the outpatient clinic at weeks 1, 2, 4, 6, and 8 after treatment had begun.

T-PEMF used in this intervention is as described in the introduction with a low electric field near 0.0004 V/m and with to a pulse frequency of 55 Hz. The impulse generator requires a chip card to be started. The card was programmed with 20 treatments. When the helmet is used, time and date for every treatment is stored on the card. Besides, it secures that the participants meet at the clinic during the intervention for new cards.

### Ethics

Referred patients were given oral and written information concerning the study before giving their written consent for participation. The investigation was conducted in accordance with the Helsinki II Declaration. The study was approved by the Scientific Ethics Committee for the Central Denmark Region, the Danish Data Protection Agency (approval number 1-16-02-337-16) and registered at ClinicalTrials.gov (unique protocol ID: 1-10-72-125-16).

### Financing

Re5 ApS has developed T-PEMF technology. Navamedic Company introduced Re5-NTS (T-PEMF) to the participating centers in Denmark and provided equipment for the project, with individual centers paying rental costs. Each DUAG center financed the project costs through their respective research funds. Navamedic and Re5 ApS did not influence the design, data analysis, or reporting of the results.

## Results

A total of 58 participants were included (20 men and 38 women) from six centers. Fifty-two participants completed the study (89.7%). One participant requested ECT as an alternative treatment option after a few days of T-PEMF treatment. One participant was hospitalized after an overdose of zolpidem as intended suicide. One participant deteriorated without suicidal thoughts after 20 days of T-PEMF treatment and discontinued treatment. One deteriorated due to too much work at home after 35 days of treatment with T-PEMF and was hospitalized. Two participants wanted a change in medication and discontinued T-PEMF treatment.

The distribution of participants across centers and characteristics of all participants in total, as well as their division according to the duration of their current depressive episode at baseline, are shown in Table 1. At baseline, the participants suffering from chronic current depressive episode (current episode >2 years) had a higher severity as measured by HAM-D_17_ as compared with participants with a nonchronic current depressive episode.

Psychopharmacological treatment at baseline is shown in Table 2. Twelve participants had tried two antidepressant drug trials in the current episode, 31 had tried 3, 7 had tried 5–6, 4 had tried 7–10, and 3 had tried more than 10 medications. Data of medications were missing for one participant. Nineteen participants were nonresponders to ECT in the current episode.

**Table 2.** Psychopharmacological treatment in patients with treatment resistant depression at baseline

Medication	Percentage/nos.	Mean dose (mg)	Range (mg)
Antidepressants			
Citalopram	1.7% (1)	40	40
Escitalopram	3.4% (2)	20	20
Sertraline	1.7% (1)	100	100
Venlafaxine	19.0% (11)	211	75–300
Duloxetine	15.5% (9)	87	60–120
Nortriptyline	25.9% (15)	108	75–150
Clomipramine	7.0% (4)	88	50–150
Moclobemide	3.4% (2)	525	450–600
Isocarboxazide	5.2% (3)	40	30–50
Mirtazapine	12.1% (7)	33	7.5–60
Agomelatine	10.3% (6)	38	25–50
Prothiadene	1.7% (1)	225	225
Vortioxetine	12.1% (7)	19	15–20
Mianserine	1.7% (1)	30	30
Amitriptyline	1.7% (1)	100	100
Augmentation			
Lithium	12.1% (7)	21 mmol	8.1–48 mmol
Lamotrigine	15.5% (9)	230	100–500
Antipsychotics			
Quetiapine	27.6% (16)	102	12.5–300
Aripiprazole	1.7% (1)	20	20
Risperidone	3.4% (2)	2	1–3
Olanzapine	5.2% (3)	8	5–15
Sedatives			
Benzodiazepines	3.4% (2)	15	15
Zoplicone	5.2% (3)	6	3.75–7.5
Melatonin	1.7% (1)	2	2
Promethazine	3.4% (2)	25	1
Total number	117		
Mean number of medications	2.02 (range 2–10)		

The mean HAM-D_17_ scores decreased from baseline (mean = 20.6, SD 4.0) to endpoint (mean = 12.6, SD 7.1; *N* = 58; *p* < 0.05; independent samples *t*-test). The mean scores on the HAM-D_6_ decreased similarly from baseline (11.2, SD 2.4) to endpoint (6.2, SD 4.1; *p* < 0.05; independent samples *t*-test).

HAM-D_17_ and HAM-D_6_ baseline scores and scores every second week of completers are presented in Table 3. There were three dropouts in each group.

**Table 3.** HAM-D_17_ and HAM-D_6_ baseline scores and weekly scores (mean and SD) for the study period in patients with treatment resistant depression stratified according to duration of depression at baseline

	*N*/*n*	HAM-D17 (mean [SD])				HAM-D6 (mean [SD])			
		Duration of the current depression				Duration of the current depression			
		Total	≤2 years	>2 years	Sign^a^	Total	≤2 years	>2 years	Sign^a^
Week									
Baseline	33/25	20.6 (4.0)	19.3 (3.7)	22.3 (3.8)	0.01	11.2 (2.4)	10.6 (2.3)	12.1 (2.2)	0.01
Week 2	32/24	15.9 (6.7)	14.0 (6.5)	18.5 (6.2)	0.01	8.8 (3.8)	7.6 (3.6)	10.4 (3.5)	0.01
Week 4	31/24	14.5 (6.8)	12.6 (6.4)	17.0 (6.6)	0.02	7.9 (4.0)	6.8 (3.8)	9.3 (3.9)	0.02
Week 6	30/23	12.6 (6.6)	11.5 (6.4)	14.0 (6.7)	0.18	7.0 (4.3)	6.1 (4.2)	8.0 (4.1)	0.10
Week 8	30/22	11.6 (6.6)	9.9 (6.3)	14.0 (6.4)	0.03	6.2 (4.1)	5.3 (4.3)	7.3 (3.5)	0.07

a Independent samples *t*-test (Levene’s Test for Equality of Variances).

Note: *N*, patients with duration ≤2 years; *n*, patients with duration >2 years; Sign, HAM-D_17_ and HAM-D_6_ ratings ≤2 years versus >2 years.

Sensitivity analyses comparing participants with nonchronic and chronic current depressive episodes were conducted. The participants suffering from a chronic current depressive episode were more severely depressed at baseline (*p* < 0.01). In participants suffering from a nonchronic depressive episode, a mean reduction of −9.4 points from baseline to the end of study on HAM-D_17_ was observed, as compared with participants with a chronic current depressive episode where a mean HAM-D_17_ reduction of −8.3 points from baseline to the end of study was observed. In participants suffering from a nonchronic depressive episode, a mean HAM-D_6_ reduction of −5.3 points from baseline to end of study was observed, as compared with participants with a chronic current depressive episode where a mean HAM-D_6_ score reduction of −4.8 points was observed.

Endpoint results for response and remission are shown in Table 4. In total, 39.7% responded on the HAM-D_17_ scale and 24.1% achieved remission. Among completers, 44.2% responded and 26.9% achieved remission.

**Table 4.** Response and remission at end of treatment (defined as HAM-D_17_ < 8 or as HAM-D_6_ < 5) and WHO-5 ≥ 50 in patients with treatment resistant depression at baseline stratified according to duration of depression

	Total/all participants (%)	Participants with a duration of depression (≤2 years)	Participants with a duration of depression (>2 years)	Sign^a^
Completers				
*N*	52	30	22	
Ham-D_17_ response	44.2%	53.3%	31.8%	0.12
Ham-D_17_ remission	26.9%	33.3%	18.2%	0.22
Ham-D_6_ response	46.2%	56.7%	31.8%	0.08
Ham-D_6_ remission	40.4%	56.7%	18.2%	0.05
WHO-5 ≥ 50	44.2%	56.7%	27.3%	0.04
All				
*N*	58	33	25	
Ham-D_17_ response	39.7%	48.5%	28.0%	0.11
Ham-D_17_ remission	24.1%	30.3%	16.0%	0.21
Ham-D_6_ response	41.4%	51.5%	28.0%	0.07
Ham-D_6_ remission	36.2%	51.5%	16.0%	0.05
WHO-5 ≥ 50	39.7%	51.5%	24.0%	0.03

a Pearson chi-square test.

Similar to previous analyses, we compared participants with a chronic versus nonchronic depressive episode showing that 48.5% of the nonchronic participants (*N* = 33) responded on the HAM-D_17_ and 30.3% achieved remission at endpoint (Table 4). Among participants (*N* = 25) suffering from a chronic depressive episode, 28.0% responded and 16.0% obtained remission on the HAM-D_17_ scale at endpoint.

At endpoint, 51.5% of participants suffering from nonchronic current depressive episode responded on the HAM-D_6_ scale and 51.5% achieved remission. Among participants suffering from chronic current depressive episode, 28.0% responded and 16.0% obtained remission on the HAM-D_6_ scale, respectively.

In general, response and remission proportion were higher in completers as compared with noncompleters.

For all participants, the mean WHO-5 increased significantly from baseline (mean 19.93, SD 15.9) to endpoint (38.0, SD 27, *p* < 0.05). Among participants with nonchronic depression, 51.5% achieved a score of WHO-5 ≥ 50 as compared with 24.0% in participants with chronic current depressive episode (Table 4). Among the completers, the proportion was even higher.

Among all the 19 ECT nonresponders, 34.6% responded and 28.6% achieved remission on the HAM-D_17_ scale at the endpoint. On the HAM-D_6,_ 33.3% responded and 28.6% achieved remission. Among the 10 ECT nonresponders who suffered from a nonchronic depression, 50.0% responded and 30.0% achieved remission on the HAM-D_17_ scale. Among the nine ECT nonresponders who suffered from a chronic current depressive episode, 33.3% responded and 11.1% achieved remission on the HAM-D_17_.

If the results were divided according to severity of the depressive episode at baseline, the response on the HAM-D_17_ was 37.5% among the minorly depressed, 53.1% among the moderately depressed, and 16.7% among the severely depressed participants. Remission on the HAM-D_17_ was achieved in 25.0% of participants suffering from a minor depression, 31.3% among participants moderately depressed at baseline, and 11.1% among participants severely depressed at baseline.

**Figure 1.**
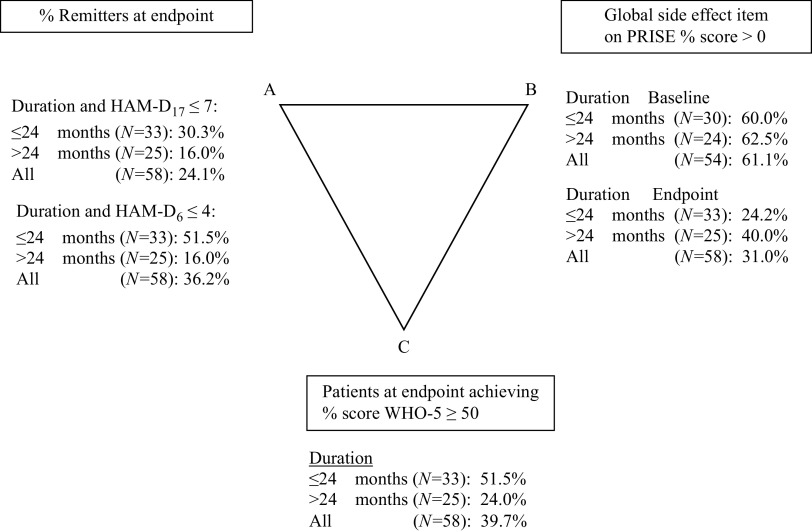
Pharmacopsychometric triangle: relationship between duration of current episode, endpoint (*N* = 58), HAM-D_17_, HAM-D_6_, side effect (PRISE), and WHO-5. Note: Baseline side effect (*N* = 54, because in four patients, baseline side effect scores were missing). Abbreviation: PRISE, Patient Reported Inventory of Side Effects.

The global side effect as measured by the PRISE decreased from 61.1% at baseline to 31.0% at endpoint. The pharmacopsychometric triangle [15, 19] illustrates the balance between the effect of pharmacotherapeutic drugs (upper left vertex A) and the induced side effects of these drugs (upper right vertex B) when taking into account self-reported well-being (lower vertex C). In the pharmacopsychometric triangle in Figure 1, the balance between symptomatic relief on HAM-D_17_ and HAM-D_6_ (endpoint), side effect on PRISE, and psychological well-being on WHO-5 is shown. The reduction in side effects was more pronounced in participants with nonchronic depression as shown in Figure 1. Among participants achieving remission, the proportion reporting side effect was reduced from 71.4 to 16.4%. Among participants not achieving remission, the proportion reporting side effect was reduced from 56.8 to 38.9%.

## Discussion

Our study supports earlier findings that a proportion of patients with treatment-resistant depression seem to benefit from add-on treatment with T-PEMF to pharmacological antidepressant treatment [8, 13, 14]. T-PEMF without treatment with antidepressants has not been investigated in this study or in the earlier studies, and it is not possible to evaluate to what extent the allowed co-medication has contributed to the findings. However, we assume that the present findings, at least not exclusively, are due to an effect of continuing with antidepressants for 8 more weeks. But we are not able to estimate the effect with our design.

Due to the very low proportion of remission in the placebo group (12.8%) in the first study [8], we decided to exclude sham T-PEMF. Among the 58 included participants with treatment-resistant depression, an overall proportion of 24% had remitted at end of treatment compared with 26.9% among the 52 completers. When remission was based on the HAM-D6, which has been shown to be more sensitive to change in severity during treatment than the HAM-D_17_ [16], the overall proportion of remitters was 36.2 and 40.4% in the total sample and completers, respectively.

The proportion of remitters was higher in participants suffering from a nonchronic depressive episode at baseline, as compared with participants suffering from a chronic depressive episode. This is in accordance with previous data showing that treatment resistance increases with the duration of the current episode [4, 5]. Despite of the lower effect observed in this patient population, there is still an indication for treatment, as no other treatment has shown a significantly better response.

The potential beneficial effect of T-PEMF was also seen in patients who were nonresponders to ECT. The indication for T-PEMF as compared with that of r-TMS and ECT thus needs to be clarified in the future. ECT seems to be more effective than rTMS for depression, especially in the short term, particularly for patients suffering from psychotic depression, severe suicidality, and lack of fluid intake [20]. ECT has, however, more side effects. Mild to moderate depressive episodes of shorter duration seem to be associated with antidepressant effect of rTMS [21]. T-PEMF treatment has the advantage that it can be used at the participant’s home due to no risk of treatment-induced seizures and ease of treatment administration, combined with a favorable side-effect profile. In our study, the side effects of the medication used were reduced considerably during treatment with T-PEMF, which might reflect an indirect effect of perceived side effects in participants.

Decades of research has been performed to elucidate the mechanism of ECT and indicates involvement of numerous biologic processes, including alterations in neuroplasticity, levels of various neurotrophic factors and neurotransmitters, functional connectivity, immune mechanisms, neuroendocrine function, as well as epigenetic processes [22]. Our study indicates that T-PEMF may have an effect in some ECT nonresponders, which may imply a different neurobiological effect.

The patient’s own assessment of improved psychological well-being during treatment is an important symptom domain, which is not captured by the HAM-D_17_ scale [4]. Therefore, we used the WHO-5 scale to measure quality of life. A score on the WHO-5 scale ≥50 has earlier been shown to correlate with remission of depression [4]. In our study, the proportion of participants achieving a WHO-5 score ≥ 50, which corresponds to no well-being problems, was in line with the HAM-D_6_ results.

The current study design, a single-arm cohort study, makes it impossible to infer treatment effect of T-PEMF per se due to an unknown proportion of spontaneous remitters and of patients remitted due to the underlying treatment as usual given over the course of the study as well as an unknown impact of expectation. However, a previous study has shown that remission rates were as low as 8% in a 2-year follow-up period in patients with treatment-resistant depression who received treatment as usual [23]. Albeit this proportion would not necessarily have been so low in the present study sample, the finding supports a low chance of remission associated with treatment as usual in TRD patients. Similarly, we were unable to control for confounders due to the single arm, nonrandomized design. Also, since the duration of the current episode was not recorded as a continuous variable, it was not possible to investigate the association between duration of the current episode and outcome beyond the duration of 2 years or less versus more than 2 years.

The current study design has some limitations. A single-arm cohort study makes it impossible to infer the treatment effect of T-PEMF per se due to an unknown proportion of spontaneous remitters as well as an unknown impact of expectation. Similarly, due to the design, we were unable to control for confounders. Furthermore, the participants received different kinds of psychotropic medicine and suffered from variation in duration and severity of episodes as well.

We conclude that despite these limitations, this study supports earlier findings that patients with treatment-resistant depression experience symptom reduction when treated with T-PEMF. Future randomized studies should further address the impact of the duration of the current episode and other potential predictors of response. Furthermore, this treatment could also be investigated for similar indications within the affective spectrum, for example, bipolar depression.

## Data Availability

We expect that data supporting the results in the article will be archived in an appropriate public repository.
